# Supplementary material for: Differential Factors for Predicting Outcomes in Left Main versus Non-Left Main Coronary Bifurcation Stenting
Source: J Clin Med. 2021 Jul 7;10(14):3024. doi: 10.3390/jcm10143024 (PMC8306985; doi:10.3390/jcm10143024)
Supplement: Supplementary file 1 [file jcm-10-03024-s001.zip › jcm-1241834-supplementary.pdf]

Supplementary Table S1. Quantitative coronary angiography analysis.

	Overall (N=2648)	Non-LMB (N=1713)	LMB (N=935)	p
Reference diameter of MV (Pre, mm)	3.24 ± 0.48	3.10 ± 0.42	3.50 ± 0.46	<0.001
Reference diameter of MB (Pre, mm)	2.88 ± 0.46	2.81 ± 0.43	3.01 ± 0.48	<0.001
Reference diameter of SB (Pre, mm)	2.60 ± 0.44	2.44 ± 0.31	2.90 ± 0.48	<0.001
Minimal luminal diameter of MV (Pre, mm)	0.86 ± 0.50	0.77 ± 0.47	1.01 ± 0.51	<0.001
Minimal luminal diameter of ostial MB (Pre, mm)	1.21 ± 0.75	1.17 ± 0.75	1.29 ± 0.76	<0.001
Minimal luminal diameter of SB (Pre, mm)	1.48 ± 0.83	1.27 ± 0.69	1.87 ± 0.90	<0.001
Minimal luminal diameter of MV (Post, mm)	2.78 ± 0.46	2.68 ± 0.44	2.95 ± 0.47	<0.001
Minimal luminal diameter of SB (Post, mm)	1.71 ± 0.81	1.41 ± 0.70	2.26 ± 0.68	<0.001
Minimal luminal diameter of ostial MB (Post, mm)	2.92 ± 0.49	2.81 ± 0.46	3.10 ± 0.49	<0.001
Lesion length of MV (mm)	18.49 ± 10.39	19.25 ± 10.23	17.09 ± 10.53	<0.001
Lesion length of SB (mm)	5.22 ± 6.68	5.61 ± 6.71	4.50 ± 6.58	<0.001
Bifurcation Angle	71.33 ± 21.94	64.16 ± 17.78	84.45 ± 22.75	<0.001
Diameter stenosis of MV (Pre, percent)	73.63 ± 14.80	75.16 ± 14.69	70.83 ± 14.60	<0.001
Diameter stenosis of SB (Pre, percent)	44.26 ± 27.18	48.36 ± 26.49	36.74 ± 26.84	<0.001
Diameter stenosis of MV (Post, percent)	15.61 ± 9.94	14.64 ± 9.78	17.38 ± 9.98	<0.001
Diameter stenosis of SB (Post, percent)	36.14 ± 26.20	43.40 ± 26.48	22.83 ± 19.64	<0.001

LMB, left main bifurcation; MV, main vessel; MB, main branch; SB, side branch.
